# Complex Soft Tissue Injuries Following Elevator Door Entrapment: A Case Report Including Surgical Management Principles

**DOI:** 10.7759/cureus.108934

**Published:** 2026-05-15

**Authors:** Abdulla A Fakhro, Hasan H Al Ajmi, Mohamed Mahmoud

**Affiliations:** 1 Plastic and Reconstructive Surgery, Houston Methodist Hospital, Houston, USA; 2 Plastic and Reconstructive Surgery, Johns Hopkins Aramco Healthcare, Dhahran, SAU

**Keywords:** avulsion laceration, elevator injury, negative pressure wound therapy, plastic surgery, reconstructive elevator, soft tissue trauma, wound closure

## Abstract

We present the case of a 28-year-old female who sustained multiple, complex soft tissue injuries, including a significant avulsion-type laceration in the gluteal region, following entrapment in an elevator door. The patient presented with a primary vertically oriented laceration measuring 21 cm over the right buttock, characterized by extensive undermining, alongside secondary lacerations to the posterior thigh and lateral malleolus. Prompt surgical intervention was performed following plastic surgery principles of careful debridement, thorough irrigation, and tension-free, layered closure. Postoperatively, the wound was managed with negative pressure wound therapy (NPWT), resulting in an excellent functional and esthetic outcome without complications at two-week and six-month follow-up. This case underscores the critical role of the plastic surgeon in the immediate and definitive management of high-energy, complex soft tissue trauma. Furthermore, we review the literature concerning elevator-related injuries and the application of the reconstructive elevator concept in managing such challenging wounds.

## Introduction

Elevator-related injuries, while statistically uncommon, frequently result in high-energy, complex trauma to the soft tissues, often involving crush, avulsion, and shearing forces [1,2]. The resulting wounds are typically contaminated and characterized by extensive tissue devitalization and undermining, presenting a significant challenge for definitive closure and functional preservation [3]. The management of these injuries requires a systematic approach, with the plastic surgeon playing a pivotal role in wound assessment, debridement, and reconstruction [4]. The plastic surgery literature on complex soft tissue injuries from this specific mechanism is notably sparse, with most case reports focusing on extremity entrapments.

This case report details the successful surgical management of a patient with multiple, deep soft tissue lacerations sustained from elevator door entrapment. Our experience highlights the importance of adhering to established plastic surgery tenets, including the timely application of the “reconstructive elevator” concept, which advocates for selecting the optimal reconstructive option to achieve the best outcome [5,6].

## Case presentation

**Figure 1:**
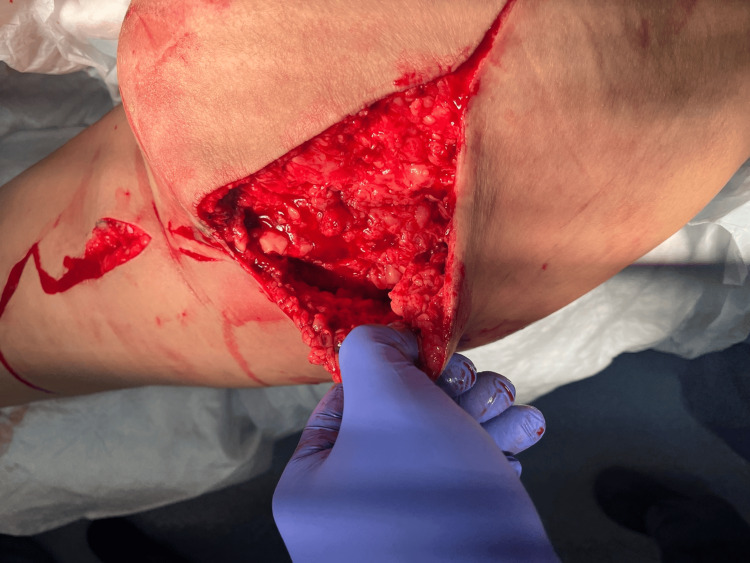
Pre-operative view of the primary gluteal laceration

**Figure 2:**
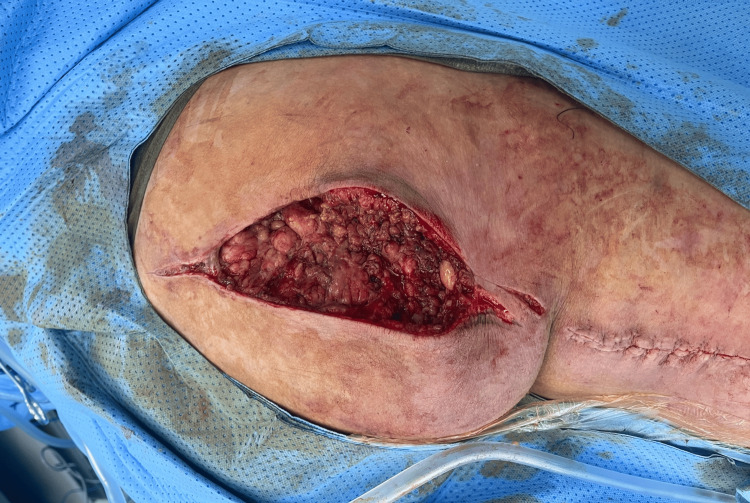
Pre-operative view of gluteal-thigh laceration

A 28-year-old female presented to the emergency department following entrapment in a moving elevator door. The mechanism of injury involved a combination of crushing and shearing forces as the door closed and the elevator car moved. The patient was conscious and hemodynamically stable upon arrival (Figures 1, 2).

The primary injury was a large, vertically oriented laceration measuring 21 cm over the right gluteal region. The wound exhibited features of an avulsion injury, with undermining extending approximately 15 cm laterally and 10 cm inferomedially. The depth extended to the deep fascia, with visible contamination and devitalized tissue at the wound edges. Secondary injuries included two longitudinal lacerations (9 cm and 5 cm) over the posterior right thigh and a 3 cm laceration over the right lateral malleolus. Associated trauma included contusions to the left wrist, left foot, and back.

**Figure 3:**
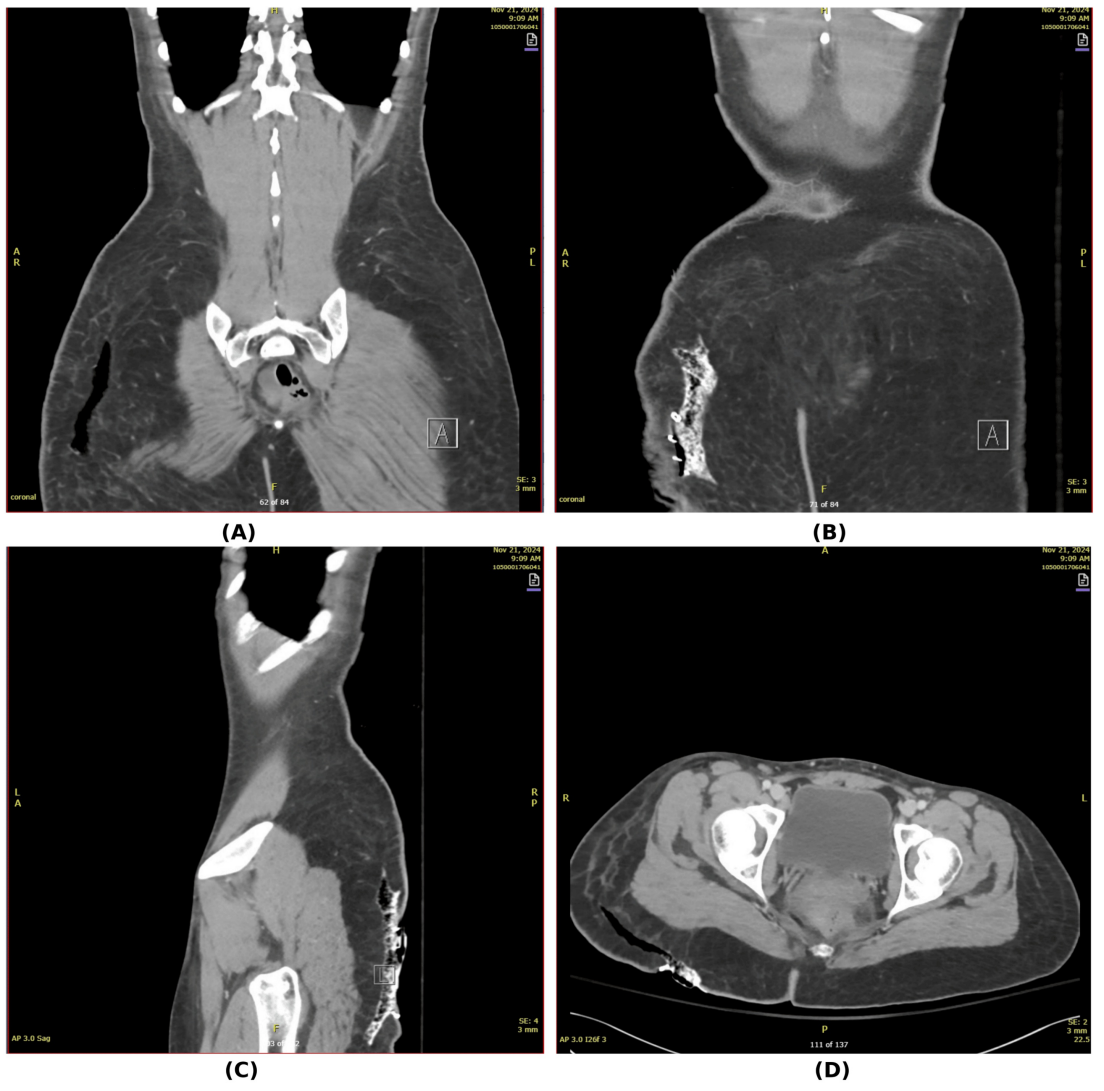
Contrast-enhanced CT of the abdomen and pelvis Contrast-enhanced CT of the abdomen and pelvis. (A) Coronal view demonstrating intact abdominal viscera and no free fluid or pneumoperitoneum. (B) Coronal image at the level of the iliac crests showing preserved gluteal musculature with no intramuscular hematoma or deep fascial disruption. (C) Sagittal image at the level of the greater trochanter confirming the superficial nature of the soft tissue injury, limited to the subcutaneous plane above the deep fascia. (D) Axial image at the level of the ischial tuberosities demonstrating intact neurovascular structures and no osseous pathology. The imaging confirms the injury extending to the level of the deep fascia only, with no evidence of vascular compromise, free air, or acute fracture.

Contrast-enhanced computed tomography (CT) of the abdomen and pelvis confirmed the superficial nature of the wound and demonstrated no evidence of vascular compromise, free air, fluid collection, or acute osseous injury (Figure 3).

Surgical management

**Figure 4:**
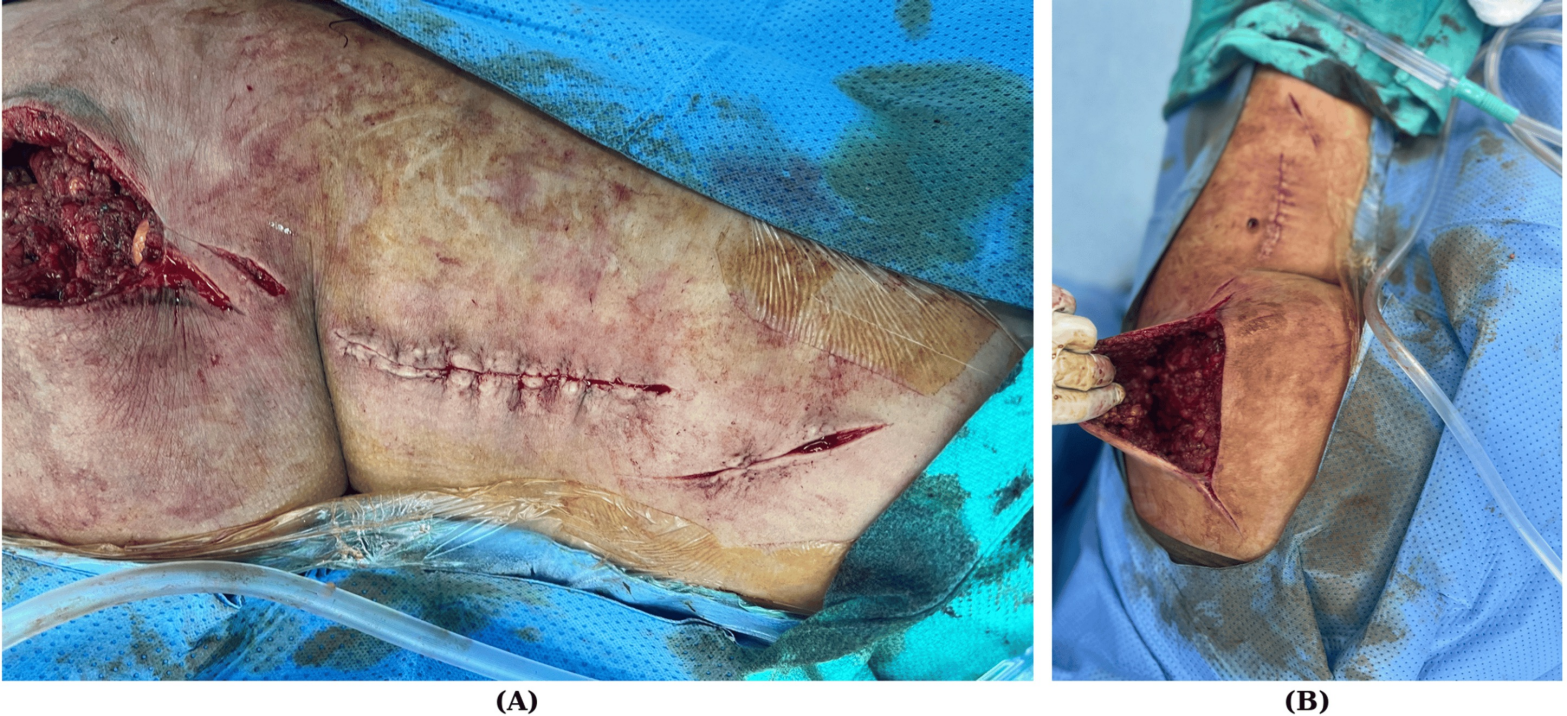
Intra-operative view following debridement Intra-operative view following systematic debridement and copious irrigation with 6 liters of normal saline. (A) The primary gluteal wound after excision of devitalized tissue, demonstrating well-vascularized wound margins with punctate bleeding and exposed deep fascia at the wound base. Note the extent of lateral undermining. (B) The wound after completion of debridement, showing clean, viable tissue margins suitable for tension-free layered closure.

The patient was taken to the operating theater for urgent wound exploration and definitive management under general anesthesia. Empiric intravenous cefazolin 2 grams was administered within 60 minutes of presentation. The principles of trauma plastic surgery guided the surgical approach and are as follows (Figure 4).

**Figure 5:**
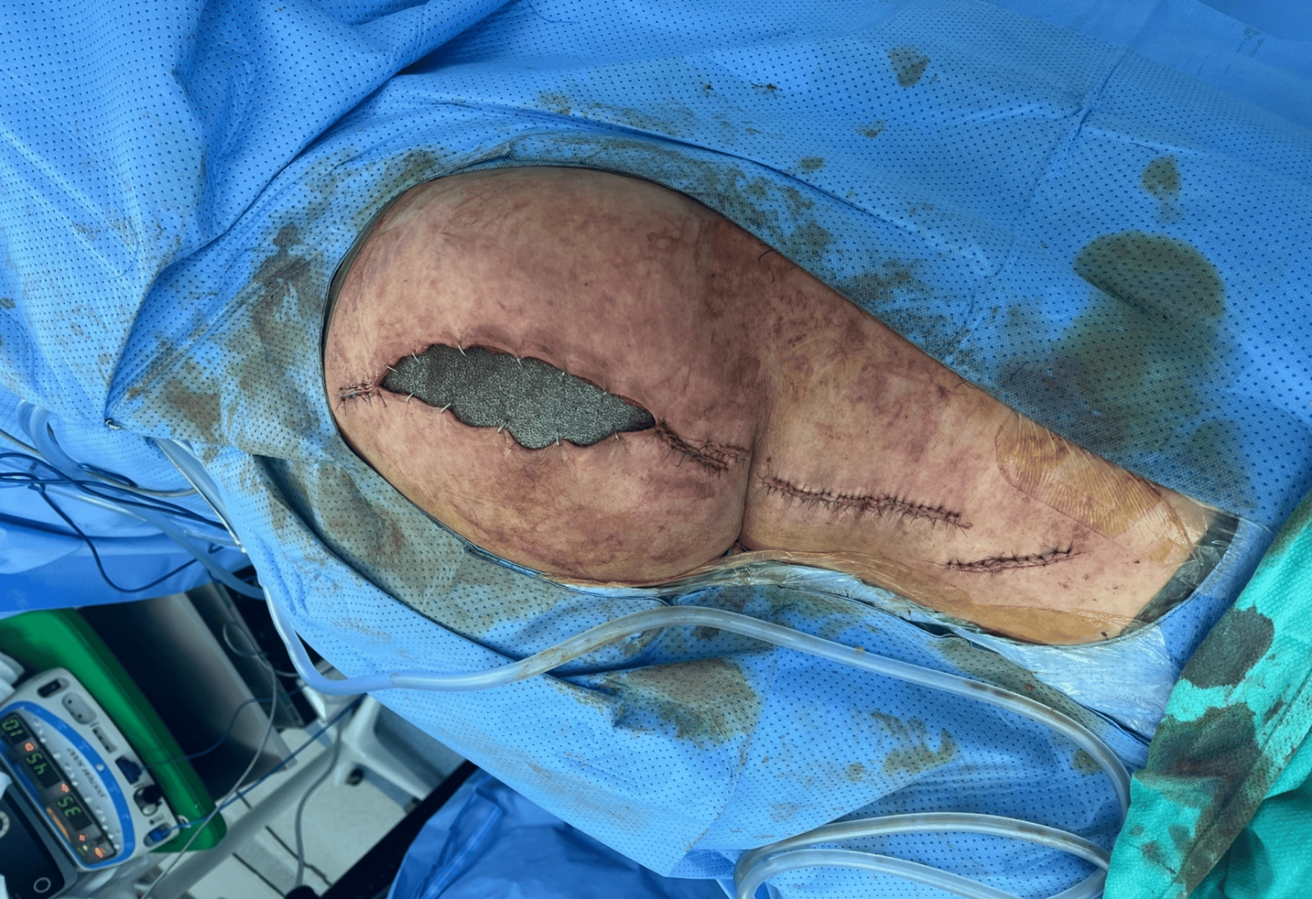
Post-operative view immediately after layered closure and application of NPWT NPWT, negative pressure wound therapy.

1. Debridement and irrigation: All devitalized, contaminated, and non-viable tissue was sharply excised until bleeding, and healthy tissue margins were achieved. Copious irrigation with 6 liters of normal saline was performed using a high-volume, low-pressure jet lavage system. Numerous debris fragments, including elevator door material and damaged fabric, were mechanically removed [7].

2. Tension-free, layered closure: A meticulous, multilayered closure was performed to minimize tension on the skin edges. The deep fascia and subcutaneous tissues were approximated using absorbable sutures (Monocryl 2-0 and 3-0) to obliterate dead space [8]. The skin was closed with non-absorbable sutures (Nylon 5-0) in an interrupted fashion.

3. Adjunctive wound care: Negative pressure wound therapy (NPWT) was applied at a continuous pressure of 100 mmHg as a bolster dressing to secure the closure, reduce postoperative edema, and promote microcirculation at potentially compromised wound margins [9] (Figure 5).

4. Antimicrobial coverage: A seven-day course of intravenous cefazolin 1 gram every 8 hours was continued postoperatively, followed by oral cephalexin 500 mg four times daily for an additional seven days. Local anesthetic infiltration of 20 milliliters of 0.25% bupivacaine was used for pain control. Tetanus prophylaxis was updated accordingly.

Postoperative course and outcome

The patient’s postoperative recovery was uneventful. The NPWT dressing was maintained for five days and then converted to a standard sterile dressing. The wound demonstrated excellent healing with no evidence of hematoma, seroma, dehiscence, or infection. Sutures were removed at two weeks, revealing a well-healed, linear scar.

Objective scar assessment was performed using the Patient and Observer Scar Assessment Scale (POSAS) [10,11]. At two weeks, the POSAS Observer Scale score was 14/60, reflecting minimal erythema, good color match, and appropriate pliability. The scar measured 21 cm in length with an average width of 3-4 mm. The patient reported a POSAS Patient Scale score of 8/60, indicating high satisfaction and minimal symptoms (visual analog scale (VAS) pain 0/10). The POSAS is used with permission and in accordance with its published guidelines. The patient reported complete, pain-free functional recovery with no limitations in ambulation or activities of daily living.

Six-month follow-up

**Figure 6:**
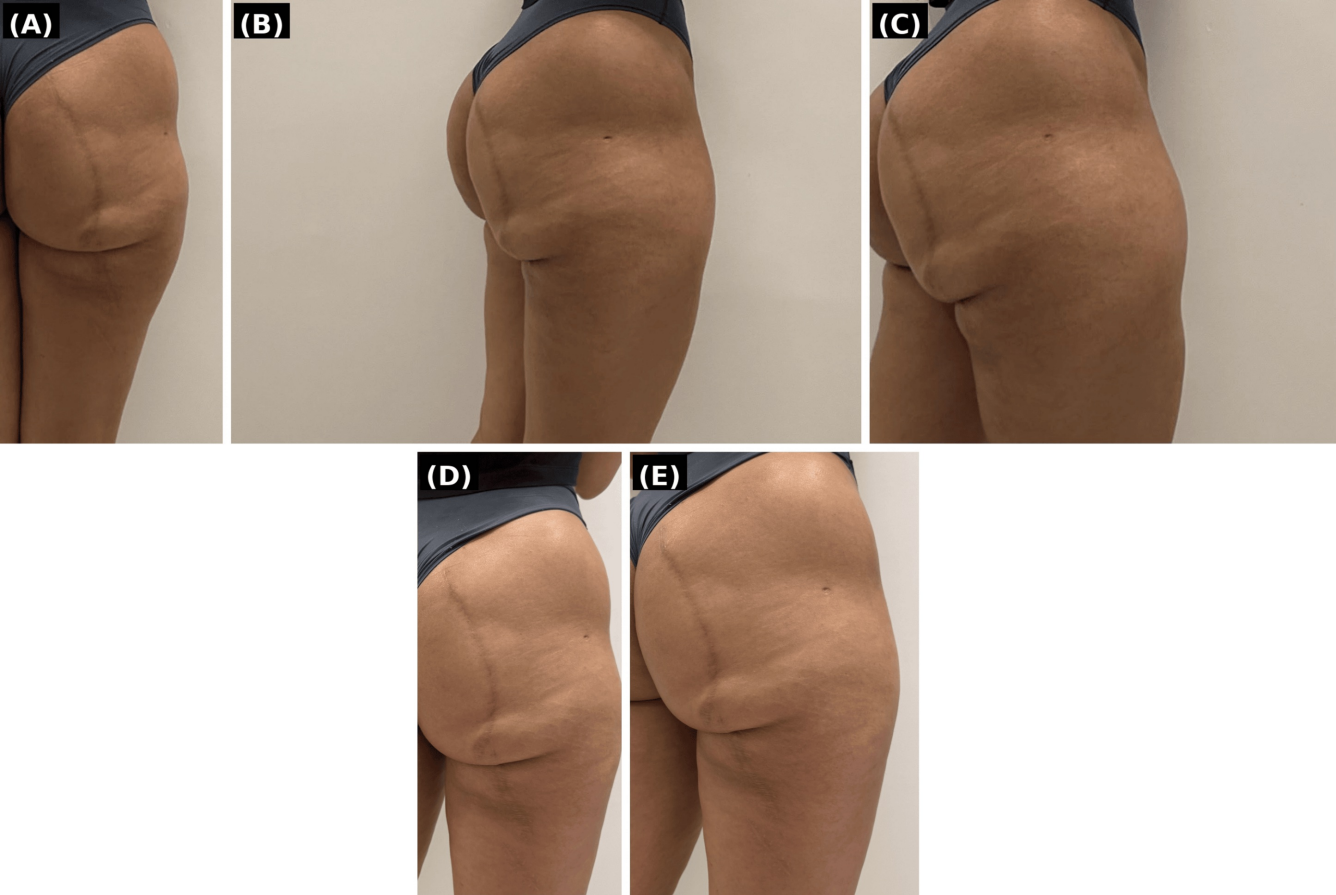
Six-month follow-up views (A) Right lateral view showing a well-integrated linear scar with minimal hyperpigmentation along the vertical axis of the right gluteal region. (B) Posterior view demonstrating overall preservation of gluteal contour with a mild contour irregularity at the site of maximum avulsion. (C) Posterolateral view showing scar pliability and soft tissue remodeling. (D) Left lateral view confirming the absence of hypertrophic scarring or keloid formation. (E) Oblique view demonstrating the overall esthetic outcome and subcutaneous volume deficit amenable to autologous fat grafting.

At six months, physical examination revealed a flat, pliable scar with minimal hyperpigmentation and no hypertrophic scarring or keloid formation. A mild contour irregularity was noted at the site of the primary laceration, consistent with subcutaneous volume loss from the initial avulsion. The patient was counselled regarding autologous fat grafting as a secondary refinement procedure. The patient maintained a full range of motion and reported high satisfaction with the overall outcome (Figure 6).

## Discussion

This case exemplifies a complex, high-energy truncal soft tissue injury in which adherence to fundamental plastic surgical principles proved critical to achieving optimal outcomes. The patient's recovery was directly attributable to emergent debridement, comprehensive irrigation, and precise tension-free layered closure that respected tissue planes and maintained vascular integrity.

Elevator accidents occur infrequently, yet cause disproportionate serious harm. O'Neil et al. reported an estimated 29,030 elevator-related injuries among children in the United States from 1990 to 2004, with soft tissue injuries being the most frequent type and the upper extremity the most commonly affected body region [1]. Similarly, McCann and Zaleski documented approximately 17,000 elevator-related injuries annually among all age groups, with the majority caused by door entrapment mechanisms [2]. More recently, Yamashita et al. (2025) conducted a forensic autopsy analysis of elevator-related fatalities, emphasizing that crush and avulsion mechanisms are the predominant causes of severe injuries and that preventive measures, including improved sensor technology, could significantly reduce such incidents [4].

Despite these epidemiological data, there is a notable gap in the plastic surgery literature regarding the management of complex soft tissue wounds from elevator door entrapment. A PubMed search using the keywords "elevator injury" AND "soft tissue" AND "gluteal" yielded seven results, of which only two specifically addressed complex truncal injuries related to elevator door entrapment. Most published reports focus on extremity, head, and neck injuries [1,2]. Truncal and pelvic soft tissue trauma from elevator door mechanisms, as in the present case, remains significantly underreported. The fundamental principles guiding management are consistent across anatomical locations: aggressive debridement, thorough irrigation, and careful tension-free reconstruction [3,5].

The avulsion mechanism in this injury caused significant damage to the subcutaneous vascular network, resulting in tissue devitalization not immediately apparent on initial inspection [12]. Boettcher-Haberzeth and Schiestl emphasized that avulsion injuries are frequently underestimated in their initial assessment, and that the extent of undermining and vascular compromise often becomes apparent only during surgical exploration [12]. This observation is consistent with our intraoperative findings, which revealed undermining extending 15 cm laterally and 10 cm inferomedially beyond the visible wound edges.

**Figure 7:**
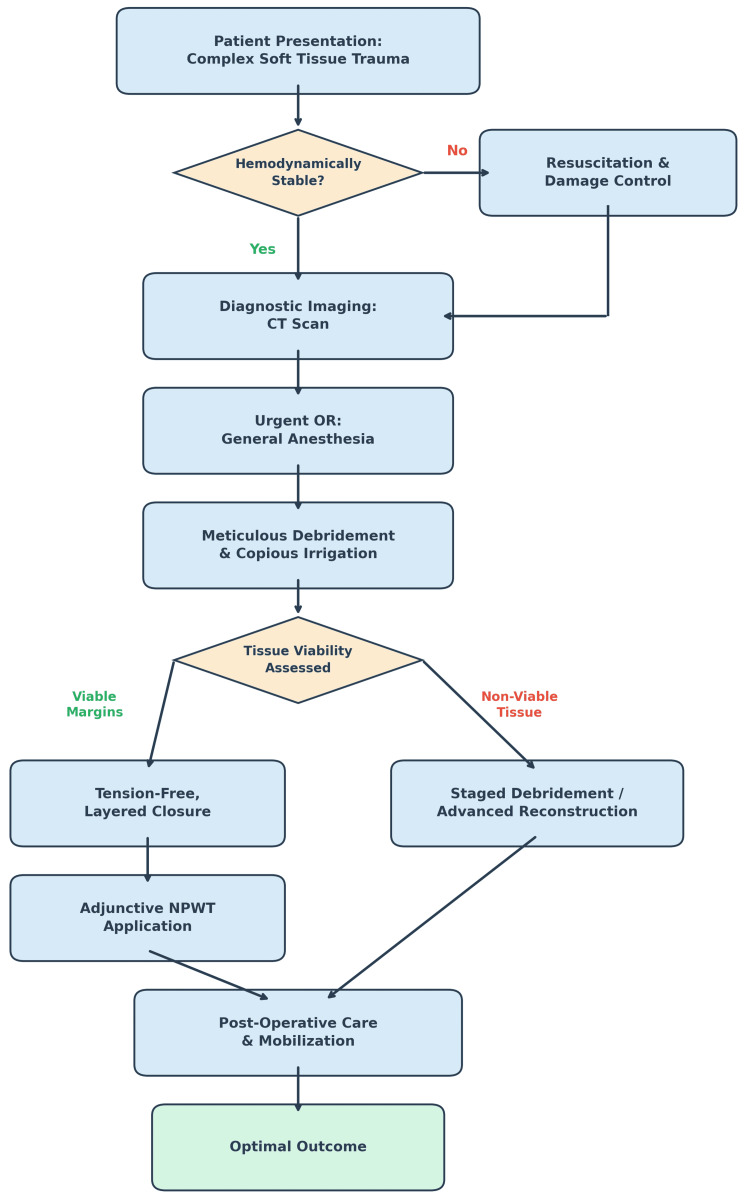
Surgical management algorithm for complex soft tissue trauma This flowchart illustrates the decision-making process from initial presentation to definitive closure and adjunctive therapy. NPWT, negative pressure wound therapy.

The gluteal region poses unique challenges for primary closure due to the constant mechanical forces generated by movement and sitting. Layered closure with deep sutures to approximate the fascia and subcutaneous tissues redistributes tension away from the skin layer, minimizing the risk of dehiscence [8]. Early, thorough debridement remains central to effective wound management in contaminated trauma. Singh et al. demonstrated in their prospective study of 130 open fractures that infection rates increase significantly when debridement is delayed beyond 24 hours, reinforcing the importance of urgent surgical intervention [13]. Attinger et al. emphasized that debridement should be performed until only healthy, well-vascularized tissue remains, and that this step is the single most important factor in optimizing wound healing outcomes [5]. In our case, debridement was performed within hours of presentation, and copious irrigation with 6 liters of normal saline was carried out in accordance with current evidence supporting volume-based lavage for contaminated traumatic wounds [7]. The systematic decision-making process from initial presentation through hemodynamic assessment, debridement, tissue viability assessment, and definitive closure is illustrated in Figure 7.

**Figure 8:**
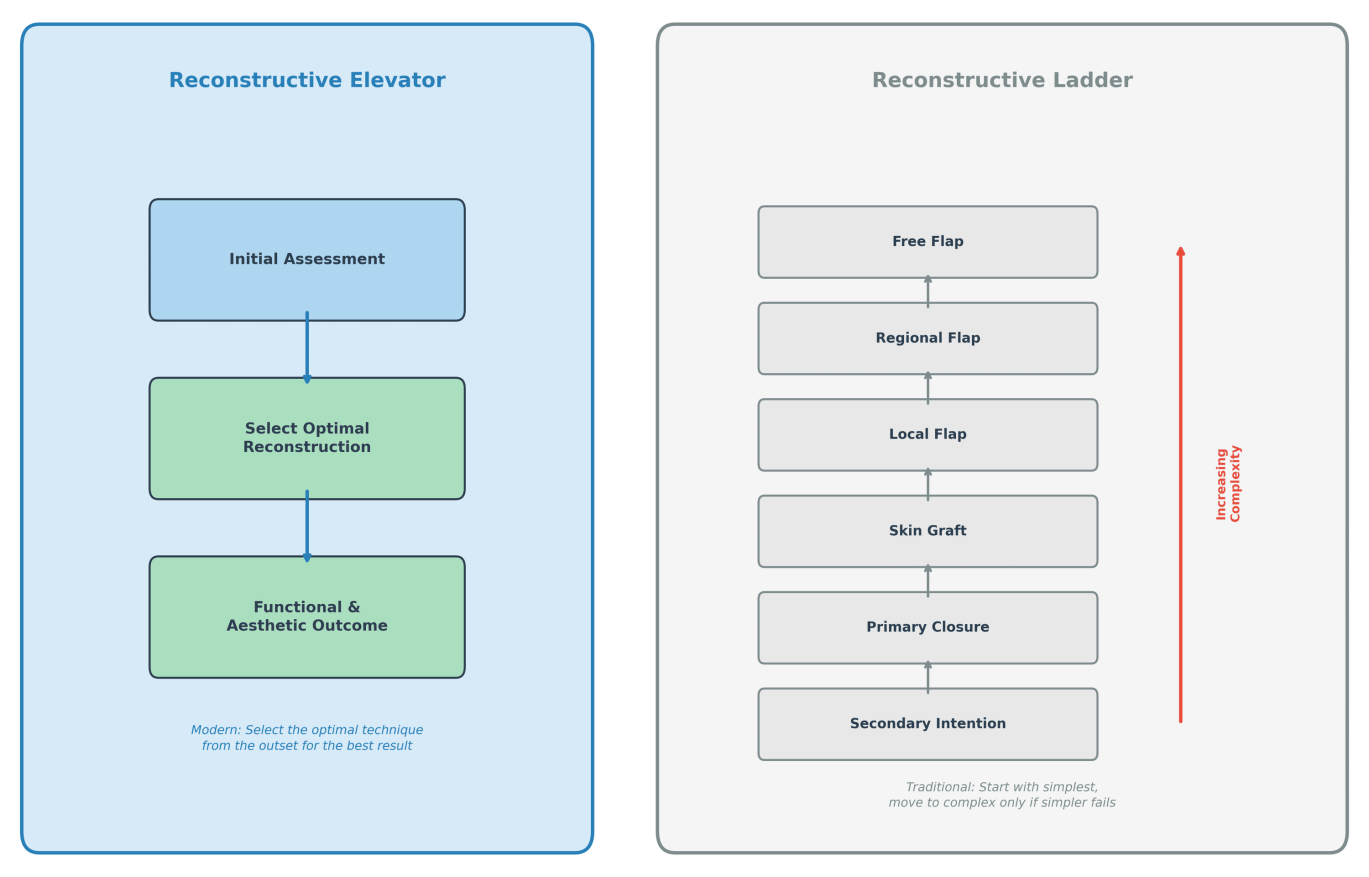
The reconstructive elevator vs. the reconstructive ladder The traditional ladder approach advocates starting with the simplest technique and escalating only upon failure, while the modern elevator approach allows immediate selection of the optimal reconstructive method.

The concept of the "Reconstructive Elevator" represents a modern evolution of the traditional "reconstructive ladder" (Figure 8) [6]. The traditional ladder advocates a stepwise approach beginning with the simplest method (secondary intention) and progressing to more complex techniques (flaps, free tissue transfer) only when simpler options fail. In contrast, the reconstructive elevator, as described by Miller and Friedrich in the context of upper extremity reconstruction, encourages immediate selection of the optimal reconstructive method based on the initial assessment of the wound, the functional requirements of the anatomical region, and the patient's overall condition [6]. In our case, the decision to perform primary layered closure with adjunctive NPWT, rather than staged debridement or delayed closure, was guided by this philosophy. 

Several alternative management strategies were considered but ultimately not pursued. Staged debridement with delayed primary closure, while appropriate for heavily contaminated wounds with questionable tissue viability, was deemed unnecessary in this case, as sharp excision achieved clearly viable, well-vascularized wound margins during the index operation. Delayed closure would have prolonged hospitalization, increased infection risk from an open wound, and subjected the patient to additional anesthetic exposure without a clear clinical benefit. Similarly, advanced reconstructive options such as local rotation flaps or skin grafting were not indicated, as the wound edges could be approximated in a tension-free manner after adequate undermining and deep tissue approximation. The decision to augment primary closure with NPWT rather than rely on conventional bolster dressings alone reflected the reconstructive elevator philosophy of selecting the optimal adjunct from the outset to minimize complication risk in a high-tension anatomical region.

The role of NPWT as an adjunct to primary closure in high-risk wounds has been well established. Argenta and Morykwas, in their landmark clinical series, demonstrated that subatmospheric pressure therapy promotes granulation tissue formation, reduces edema, and enhances local blood flow [9]. Morykwas et al. confirmed these findings in animal models, showing a fourfold increase in blood flow and significantly accelerated granulation tissue formation at 125 mmHg of continuous negative pressure [14]. Blume et al., in a multicenter randomized controlled trial comparing NPWT with advanced moist wound therapy in diabetic foot ulcers, demonstrated significantly higher rates of complete wound closure in the NPWT group [15]. In our case, NPWT served as a bolster dressing that provided uniform mechanical support to the closure, reduced interstitial edema, and promoted microcirculatory perfusion at potentially compromised wound margins.

Scar outcome assessment using the POSAS confirmed an excellent result, with an Observer Scale score of 14/60 and a Patient Scale score of 8/60 at two weeks [10,11]. The patient maintained full functional recovery and high satisfaction at the six-month follow-up, with no evidence of hypertrophic scarring or keloid formation.

## Conclusions

This case demonstrates that complex soft tissue injuries resulting from elevator door entrapment can be effectively managed through a systematic approach grounded in plastic surgical principles. The combination of urgent, thorough debridement, tension-free layered closure, and adjunctive NPWT achieved an excellent functional and esthetic outcome, as confirmed by favorable POSAS scores and sustained patient satisfaction at six-month follow-up.

The application of the reconstructive elevator philosophy, which involves selecting the optimal reconstructive strategy from the outset rather than defaulting to the simplest option, proved instrumental in achieving primary wound healing in a high-tension anatomical region without the need for staged procedures or advanced flap reconstruction. This case contributes to the limited body of literature on truncal elevator-related injuries and highlights the critical role of the plastic surgeon in the multidisciplinary management of high-energy civilian trauma. Future prospective studies comparing primary closure with NPWT to alternative wound management strategies in high-tension truncal injuries would help establish evidence-based guidelines for this underreported injury pattern.
